# The ectoparasites and gastrointestinal helminths associated with Smith’s bush squirrel (*Paraxerus cepapi*) in South Africa

**DOI:** 10.1017/S0031182025101261

**Published:** 2026-01

**Authors:** Inge Raubenheimer, Conrad A. Matthee, Alexandr Stekolnikov, Jeanette Wentzel, Lourens Swanepoel, Sonja Matthee

**Affiliations:** 1 Department of Conservation Ecology and Entomology, Stellenbosch University, Matieland, South Africa; 2 Evolutionary Genomics Group, Department of Botany and Zoology, Stellenbosch University, Matieland, South Africa; 3 Laboratory of Parasitic Arthropods, Zoological Institute of the Russian Academy of Sciences, Saint Petersburg, Russia; 4 Hans Hoheisen Wildlife Research Station, Department of Veterinary Tropical Diseases, University of Pretoria, Onderstepoort, South Africa; 5 Centre of Veterinary Wildlife Research, Faculty of Veterinary Science, Onderstepoort, South Africa; 6 SARChI Chair in Biodiversity Value and Change, Faculty of Science, Engineering and Agriculture, University of Venda, Thohoyandou, South Africa

**Keywords:** ectoparasite diversity, Gastrointestinal helminths, *Paraxerus cepapi*, Smith’s bush squirrel, South Africa

## Abstract

*Paraxerus cepapi* is an arboreal tree squirrel that occurs in the Savanna biome of Africa, and information on its parasite diversity is limited and mostly qualitative. The aim of the study was to record the diversity and abundance of ecto- and helminth parasites associated with *P. cepapi* across its distribution in South Africa. *P. cepapi* individuals (*n* = 94) were opportunistically obtained from eight localities during 2020 to 2024. In total, 21 parasite species (19 ectoparasites and two nematodes) and one tick species group were identified. This included lice, ticks, fleas, a mesostigmatic mite, chiggers, nematodes and cestodes. Nematodes were the most prevalent (93·67%), followed by lice (80·85%). *Syphatineria cepapi* was recorded in 92·41% of *P. cepapi*, while an unknown *Strongyloides* species, resembling *S. robustus*, was recorded in 21·52% of squirrels. The lice species displayed variation in parasitope preference, while chiggers were primarily recorded in the ears. This study provides new country records for the lice species *Werneckia paraxeri* and *Enderleinellus heliosciuri*, for the chigger species *Microtrombicula polymorpha,* and for the nematode *S*. cf. *robustus*. New locality records were documented for the nematode *S. cepapi* in South Africa, and *P. cepapi* is a new host record for the eight chigger species and *S*. cf. *robustus*. It is evident that *P. cepapi* in South Africa hosts a considerably larger diversity of parasite taxa than previously recorded. Nematode counts were related to host length. These findings warrant future studies on the parasite diversity of *P. cepapi* in Africa.

## Graphical abstract

**Figure:**
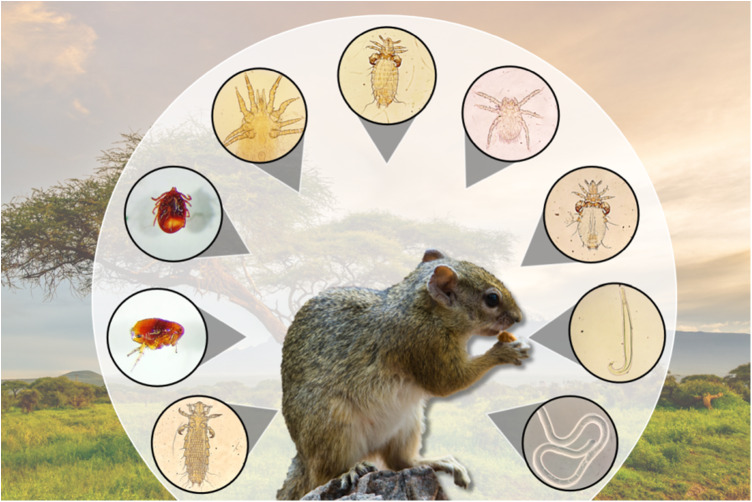

## Introduction

Smith’s bush squirrel (*Paraxerus cepapi*) is a partially arboreal diurnal tree squirrel endemic to Sub-Saharan Africa (Kingdon, 1997; Pappas and Thorington, 2013; Monadjem et al. 2015). Currently, ten subspecies are recognized, of which *P. cepapi* is distributed throughout the north and north-eastern regions of South Africa as well as regions in Namibia, southern Botswana and Zimbabwe (Thorington and Hoffmann, 2005; Thorington et al. 2012). The squirrel is typically observed in woodland habitats consisting of mopane, *Colophospermum mopane*, and *Vachellia* (previously known as *Acacia*) trees, as the old, hollowed branches of these trees act as ideal breeding and nesting areas (Linzey and Kesner, 1997a; Skinner and Chimimba, 2005). Within these habitat types, *P. cepapi* can occur in large numbers, and a localized study in mopane woodland reported that they can constitute more than 80% of the regional small mammal biomass (Linzey and Kesner, 1997b).

This uniquely social tree squirrel lives in family groups of five individuals on average (Viljoen, 1977a). The family group not only shares the nest but also partakes in allo- and autogrooming, group foraging and territory protection (Viljoen, 1977a; Skinner and Chimimba, 2005). Depending on food availability and season, its diet consists of various fruits, seeds, flowers and insects foraged from the ground and trees (Viljoen, 1977b; De Graaf, 1981; Skinner and Chimimba, 2005; Thorington et al. 2012). Ground foraging generally occurs in areas where tall and dense vegetation is absent (Viljoen, 1977b). Nests in tree cavities are lined with leaves and grass, which are frequently removed and replaced (Thorington et al. 2012). *P. cepapi* can be considered opportunistic due to its successful adaptation to anthropogenic habitats. For example, the squirrel is often observed near human settlements and often damages the roofs and electrical wiring of houses (Banotai et al. 2023). Behavioural characteristics such as their social structure, diet, nest type and use of various habitats can influence the degree of exposure to a variety of parasites (Hillegass et al. 2008; Lucatelli et al. 2020).

Current literature on the parasites associated with *P. cepapi* is limited to parasite-host monographs (Ledger, 1980; Durden and Musser, 1994; Segerman, 1995; Horak et al. 2018) and a single study conducted on *P. c. cepapi* in South Africa in 1973/74 (Viljoen, 1977c). These literature sources are often limited in their sample size and span small geographic areas, and they also lack quantitative data on all associated ecto- and helminth parasites. According to the literature, *P. cepapi* acts as a host for 13 parasite species (one nematode, one mesostigmatic mite, one unidentified trombiculid mite, one unidentified hypopi of sarcoptiform mites, two fleas, four lice and three ticks) (Viljoen, 1977b, 1977c; Ledger, 1980; Hugot, 1981; Durden and Musser, 1994; Segerman, 1995; Horak et al. 2018). However, given the squirrel’s geographic range and opportunistic nature, it is predicted that this parasite diversity is underestimated. Further, little is known about potential factors that can drive parasite infestations in the squirrel. It is possible that parasite infestations can be related to one or more host-related factors such as age, sex and reproductive state (Morand and Poulin, 2002; Morand et al. 2006; Kamiya et al. 2014), and this is also in need of further investigation.

In order to address the lack of information on the parasite diversity associated with *P. cepapi*, and more specifically the subspecies *P. c. cepapi*, squirrels were opportunistically sampled at multiple localities across their distribution in the Savanna biome of South Africa and all macroparasites were recorded. The aim of the study was to provide quantitative data on the ecto- and helminth parasite diversity that is associated with *P. cepapi* in South Africa and to explore potential host factors that can affect parasite infestations.

## Materials and methods

### Study area and sample collection

**Figure 1.**
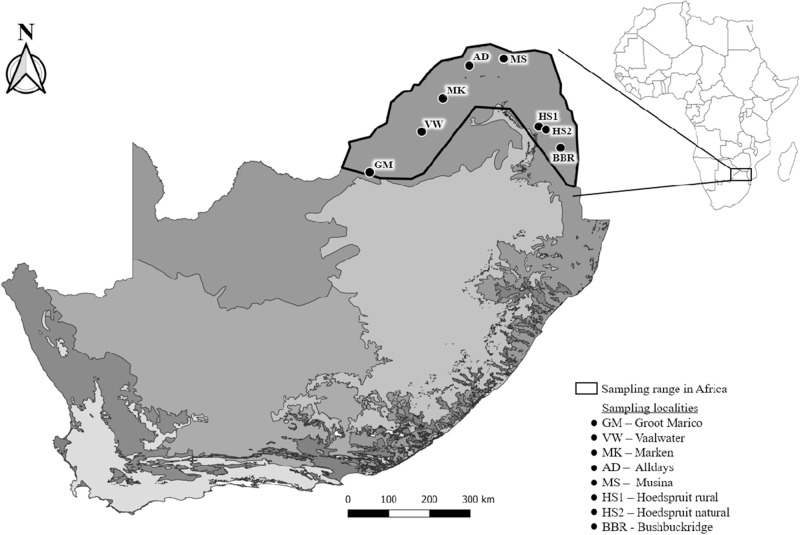
Localities (*N* = 8) where *Paraxerus cepapi* were obtained in the Savanna biome (2020–2024). Black outline represents the distribution range of the squirrel in South Africa. Locality codes correspond to Table 1. The various biomes of South Africa are indicated by the different shades of grey and obtained from openAFRICA (2015).

A total of 94 *P. cepapi* individuals were opportunistically collected (road kills, culling and traps) from eight localities in the Savanna woodlands of Limpopo, Mpumalanga and the North-West provinces of South Africa during 2020 to 2024 (Table 1; Figure 1). The localities included reserves, farms, and rural areas. Samples were collected throughout the year. Locality information and collection date were obtained for each sample. Collected individuals were individually frozen in labelled plastic bags at −20°C.

**Table 1.** Sampling localities (*n* = 8), together with codes and geographic coordinates, sampling year and number of *Paraxerus cepapi* collected from the Savanna biome of South Africa during 2020–2024

Locality	Code	Geographic coordinates		Sampling year	Sample size
Groot Marico	GM	25°35′5·87″S	26°24′24·87″E	2020−2021	3
Vaalwater	VW	24°29′6·04″S	27°48′51·63″E	2020	2
Marken	MK	23°35′20″S	28°23′6·82″E	2021−2023	13
Alldays	AD	22°41′56·93″S	29°6′1·57″E	2021	7
Musina	MS	22°22′19·23″S	29°57′35·34″E	2023	4
Hoedspruit rural	HS1	24°25′41·36″S	31°10′22·96″E	2023	40
Hoedspruit natural	HS2	24°20′45·96″S	3058′23·88″E	2021−2023	23
Bushbuckridge	BBR	24°55′16·68″S	31°33′35·28″E	2023−2024	2
**Total**					**94**

### Host examination and parasite removal

Prior to parasite removal, squirrel carcasses were thawed and systematically examined for all ectoparasites using fine-point forceps and a Leica MZ75 stereomicroscope. All ectoparasites (lice, ticks, fleas and mesostigmatic mites), apart from chiggers (trombiculid mites), were removed, counted and preserved in tubes filled with 100% ethanol. For the chiggers, only a subsample of chiggers was collected, and the parasitope (preferred attachment site on the host) was recorded. All the chiggers that were collected per parasitope were placed in the same sample tube. In addition, the distribution of individual louse species across three body regions (head, dorsal or ventral) of the host body was recorded for a subsample (*n* = 28) of the hosts. All the lice that were collected per parasitope were placed in the same sample tube.

After the removal of ectoparasites, the squirrels were dissected to remove the gastrointestinal tract (GIT). All helminths were removed from the stomach, small intestine, caecum and large intestine by placing each organ in a petri dish and examining the contents and organ wall using fine-point forceps and a stereomicroscope. All helminths were stored in 100% ethanol. The parasitope of the individual helminth species was recorded.

Information pertaining to the host, such as sex, weight, total length, hind foot length and reproductive stage, was recorded, with the exception of road kills, as their condition did not allow for such information to be collected. For males, the reproductive stage was classified as scrotal or non-scrotal depending on the visibility of the testicles on the exterior of the squirrel. For females, the reproductive stage was classified as perforated or non-perforated vagina, as well as pregnant or non-pregnant for perforated females. Pregnancy was determined during dissection.

### Parasite processing and identification

The different parasite groups were prepared for identification as follows. The lice were separated into morphospecies, after which a subsample (two males and two females per locality) of each morphospecies was cleared in lactic acid and mounted in polyvinyl alcohol (PVA) on microscope slides. A second subsample of each morphospecies was kept for molecular analysis, not reported herein. The immature life stages of lice remained undifferentiated and were reported as nymphs, and counts were presented per species. A subsample of chiggers and the mesostigmatic mite were slide-mounted in PVA, while the ticks and fleas remained unmounted in 100% ethanol. In the case of nematodes, a subsample (ten males and ten females per locality) was temporarily mounted in lactophenol, and another subsample (two females per locality) was kept for molecular identification not reported herein. Cestodes remained unmounted in 100% ethanol.

Ectoparasites and nematodes were identified to genus level or, where possible, to species level using various taxonomic reference keys. Identification of lice was done following Cummings (1912), Ferris (1919) and Werneck (1947). Tick identification followed Hoogstraal and El Kammah (1974), Walker et al. (2000), Apanaskevich et al. (2007) and Horak et al. (2018), while the two female fleas were identified to genus level following Segerman (1995). Various taxonomic references (e.g., Till, 1963; Herrin and Tipton, 1976) were used, and expert taxonomists (Eddie Ueckermann, North-West University, South Africa and Wayne Knee, Canadian National Collection of Insects, Arachnids and Nematodes, Canada) assisted with the identification of the single female mesostigmatic mite specimen. Chigger identification followed Stekolnikov (2018) together with various taxonomic literature on African chiggers reviewed therein and Stekolnikov and Matthee (Stekolnikov and Matthee 2019, 2022). Nematodes, and particularly members of the genus *Syphatineria*, previously *Syphacia*, were identified according to Sandground (1933), Hugot (1981); Anderson et al. (2009), and Gibbons (2010). The species diagnosis of the recorded *Strongyloides* species was done based on parasitic females that were morphologically and morphometrically compared to four *Strongyloides* species – of which three species (*S. fuelleborni, S. ratti* and *S. stercoralis*) have previously been recorded in Africa (Pampiglione and Ricciardi, 1972; Genta, 1989; Julius et al. 2017) and one species (*S. robustus*) occurs in tree squirrels in North America and Europe (Bavay, 1877; Linstow, 1905; Sandground, 1925; Little, 1966; Speare, 1986; Bartlett, 1995). The two cestode individuals remained unidentified.

### Data analysis

Descriptive statistics of the parasites were performed following Bush et al. (1997). For each taxonomic group, as well as for each identified species within the group, the mean abundance was calculated as the sum of individuals in the taxonomic group and/or species divided by the total number of hosts that were examined, regardless of parasite presence. Prevalence was calculated as the sum of hosts, per taxonomic group and/or species within the group, that had one or more parasite individuals present, divided by the total number of examined hosts.

The relationship between parasite infestation and host factors (body size – total body length as proxy, sex, reproductive state and the interaction between sex and reproductive state) was assessed for the most prevalent and abundant parasite taxa (lice and nematodes). Total counts per parasite taxon were calculated for each host individual. Lice count data were modified (log + 1 transformed) prior to analyses to reduce variation and overdispersion in the data. Generalized linear mixed-effect models (GLMM) were constructed for lice counts following a Poisson distribution (Zuur et al. 2009) with the *vegan* package (Oksanen et al. 2007) in R (R Core Team, 2023) using year, site and month as random factors (exploratory analysis based on AIC suggested a stronger support for the Poisson distribution compared to a negative binomial distribution). This was followed by model selection using the *drop1* function (which drops every non-significant variable, i.e., does not influence the final model). For the nematode count, the data were log + 1 transformed, and thereafter a generalized linear model (GLM) with a Poisson distribution was used (the random factors were not included due to zero variance). The removal of an extreme outlier (a squirrel with 1062 nematodes) reduced the AIC from 344·9 to 339·7. The *drop1* function was again used for model selection.

## Results

In total, 21 parasite species and one tick species group were identified, of which 19 species were ectoparasites and two were nematodes (Table 2; Supplementary Table 1). Three louse species (*Johnsonpthirus heliosciuri, Werneckia paraxeri* and *Enderleinellus heliosciuri*) were recorded (Table 2). *J. heliosciuri* was the most prevalent (73·40%), followed by *W. paraxeri* (64·89%) and *E. heliosciuri* (53·19%). However, of the three species, *W. paraxeri* was the most abundant (14·32 ± 14·12) (Table 2). All three louse species co-occurred at five of the eight localities, and *J. heliosciuri* was recorded at seven of the eight localities (Supplementary Table 2). Adult lice were more prevalent than the nymph life stage for *W. paraxeri* and *E. heliosciuri*, while the opposite was recorded for *J. heliosciuri* (Table 2). Adult females appear to be more prevalent compared to males in all three louse species (Table 2). From the parasitope records (head, dorsal and ventral), *J. heliosciuri* was the most common in the dorsal region, while *E. heliosciuri* was more common in the head region, and *W. paraxeri* was equally common in all three parasitopes (Table 3).

**Table 2.** Infestation parameters of ecto- and helminth parasites recorded on *Paraxerus cepapi* (*n* = 94) in the Savanna biome, South Africa (2020–2024)

Taxa/Species	Total no. of individuals	Adults (%)	Larvae (%)	Nymphs (%)	Sex ratio (%) ♂:♀	Mean abundance (± SE)	Prevalence (%)
**Lice**	**3419**	**64**·**61**	–	**35**·**19**	**0**·**26:0**·**38**	**36**·**36 ± 5**·**25**	**80**·**85**
*Johnsonpthirus heliosciuri*	1308	28·29	–	71·71	0·12:0·16	13·92 ± 2·73	73·40
*Werneckia paraxeri* ^L^	1346	86·55	–	13·22	0·32:0·54	14·32 ± 14·12	64·89
*Enderleinellus heliosciuri* ^L^	765	88·10	–	11·37	0·40:0·48	8·14 ± 1·68	53·19
**Ticks**	**633**	–	**72**·**51**	**27**·**49**	–	**6**·**88 ± 1**·**90**	**48**·**94**
*Amblyomma* sp.	3	–	–	–	–	0·03 ± 0·02	2·13
*Haemaphysalis elliptica/ leachi* group	7	–	100·00	–	–	0·08 ± 0·07	1·06
*Haemaphysalis zumpti*	14	–	92·86	7·14	–	0·14 ± 0·06	7·45
*Rhipicephalus* cf. *simus*	2	–	–	100·00	–	0·02 ± 0·01	2·13
*Rhipicephalus* cf. *theileri*	21	–	57·14	42·86	–	0·22 ± 0·13	3·19
*Rhipicephalus* cf. *zambeziensis*	580	–	72·07	27·93	–	6·17 ± 1·88	40·43
Unknown	7	–	100·00	–	–	0·07 ± 0·06	2·13
**Fleas**	**2**	**100**·**00**	–	–	**0:100**	**0**·**02 ± 0**·**01**	**2**·**13**
*Xenopsylla* sp.	2	100·00	–	–	0:100	0·02 ± 0·01	2·13
**Mites**	**1**	**100**·**00**	–	–	**0:100**	**0**·**01 ± 0**·**01**	**1**·**06**
*Echinonyssus transvaalensis*	1	100·00	–	–	0:100	0·01 ± 0·01	1·06
**Chiggers**	–	–	**100**·**00**	–	–	–	**62**·**80**
*Ascoschoengastia ueckermanni* ^H^	–	–	100·00	–	–	–	2·13
*Microtrombicula polymorpha* ^HL^	–	–	100·00	–	–	–	3·19
*Herpetacarus decasetosus* ^H^	–	–	100·00	–	–	–	1·06
*Herpetacarus octosetosus* ^H^	–	–	100·00	–	–	–	2·13
*Hypotrombidium* sp.^H^	–	–	100·00	–	–	–	4·26
*Microtrombicula graphiuri* ^H^	–	–	100·00	–	–	–	27·66
*Microtrombicula squirreli*	–	–	100·00	–	–	–	24·47
*Schoutedenichia morosi* ^H^	–	–	100·00	–	–	–	10·00
*Walchia africaeaustralis*	–	–	100·00	–	–	–	1·06
**Nematodes**	**11 253**	**100**·**00**	–	–		**71**·**22 ± 15**·**87**	**93**·**67**
*Strongyloides* cf. *robustus* ^HL^	141	100·00	–	–	0:100	1·78 ± 0·51	21·52
*Syphatineria cepapi* ^L^	11 112	100·00	–	–	0·38:0·62	140·66 ± 19·54	92·41
**Cestodes**	**2**	–	–	–	–	**0**·**03 ± 0**·**02**	**2**·**53**
Unknown	2	–	–	–	–	0·03 ± 0·02	2·53

H New host record.

L New locality record.

HL New host and locality record.

**Table 3.** Prevalence (%) of louse species per parasitope on *Paraxerus cepapi* (*n* = 28) obtained from five localities in the Savanna biome, South Africa (2021; 2023–2024)

	Head	Dorsal	Ventral
*Johnsonpthirus heliosciuri*	14·28	46·43	39·30
*Werneckia paraxeri*	31·25	34·38	34·37
*Enderleinellus heliosciuri*	63·63	22·72	13·63

Five tick species and one species group were recorded (Table 2). *Rhipicephalus* cf. *zambeziensis* was the most prevalent (40·43%), followed by *Haemaphysalis zumpti* (7·45%) and *Rhipicephalus* cf. *theileri* (3·19%) (Table 2). Furthermore, larvae were more common (72·51%) compared to nymphs (27·49%), and no adult life stages were recorded (Table 2). Seven unidentified individuals (due to damage to mouth parts) were recorded at Musina (Supplementary Table 2).

Two flea individuals (both females) from the genus *Xenopsylla* and one adult female mite, *Echinonyssus transvaalensis*, were recorded (Table 2).

Chiggers were present on 62·80% of the squirrels (Table 2) and were recorded at five of the eight localities (Table 4). *Microtrombicula graphiuri* was the most prevalent species (27·66%), followed by *Microtrombicula squirreli* (24·47%) (Table 2). *Microtrombicula squirreli* occurred at four of the eight localities (Supplementary Table 2). Nine chigger species were recorded at Hoedspruit rural (Supplementary Table 2). Chiggers mainly occurred in the ear of the hosts (Table 4).

**Table 4.** Prevalence (%) and parasitope preference of chiggers on *Paraxerus cepapi* (*n* = 94) per sampling locality in the Savanna biome, South Africa (2020–2024)

Locality	^*^N	Prevalence (%)	Parasitope
Groot Marico	3	33·30	Ear
Vaalwater	2	–	–
Marken	13	–	–
Alldays	7	–	–
Musina	4	75·00	Ear
Hoedspruit rural	40	70·00	Ear, neck, dorsal and ventral
Hoedspruit natural	23	60·90	Ear
Bushbuckridge	2	100·00	Ear
**Total**	**94**	**51**·**1**	

* *N* = total number of host individuals per locality.

*Syphatineria cepapi* was the most prevalent (92·41%) and widespread nematode (occurred at 7 localities) and was predominantly found in the caecum (70·87%) (Table 2; Supplementary Table 2). Based on the morphological and morphometric assessment, *S*. cf. *robustus* was the most plausible taxonomic identification. *Strongyloides* cf. *robustus* occurred in 21·52% of the squirrels (in the small intestine) and was only recorded at the 2 Hoedspruit localities (Table 2; Supplementary Table 2). Only adult females were recorded (Table 2). The two unidentified cestodes were recorded at different localities and different parasitopes in the gastrointestinal tract (stomach and small intestine).

Assessment of the relationship between louse and nematode abundance, respectively, and host factors revealed no significant relationship between the number of louse individuals and any of the host factors. However, the number of nematode individuals was significantly related only to host total length (host size), with larger individuals harbouring more nematodes (Table 5).

**Table 5.** Summary of the final selected generalized linear mixed-effect model (lice) and generalized linear model (nematodes) with a Poisson distribution on the effect of host total length (TL) on the louse and nematode abundance on *Paraxerus cepapi* (*n* = 94) in the Savanna biome, South Africa (2020–2024). Bold text indicates significant responses

Taxon	Variable	Estimate	SE	*z*-value	*P*-value
Lice	TL	0·012	0·022	0·518	0·605
Nematodes	TL	0·036	0·016	2·318	**0**·**021**

## Discussion

*P. cepapi*, and more specifically *P. c. cepapi*, in South Africa hosts a relatively large diversity of parasite taxa, of which lice were the most prevalent ectoparasite group and nematodes the most prevalent helminth group. The sucking louse, *J. heliosciuri* is a recognized louse species of *P. cepapi* and other tree and bush squirrels in Africa (*Funisciurus* and *Paraxerus*) (Ferris, 1919; Viljoen, 1977c; Durden and Musser, 1994). The louse was originally described from the red squirrel, *Paraxerus palliatus,* in Kenya (Cummings, 1912) and later recorded from several other *Paraxerus* species distributed across Africa, including Namibia (Johnson, 1960) and South Africa (Durden and Musser, 1994). The species’ occurrence on African squirrels is characteristic of members of the genus *Johnsonpthirus* (Kim and Adler, 1982; Durden and Musser, 1994). It is important to note that recent genetic studies have indicated cryptic speciation among conspecific lice occurring on different rodent species (du Toit et al. 2013; Bothma et al. 2019), and more in-depth comparative research is required to fully understand the taxonomy of *J. heliosciuri* on *P. cepapi* (Martinu et al. 2015).

The present study recorded two additional louse species, *W. paraxeri* and *E. heliosciuri*, that belong to the family Enderleinellidae and are exclusive parasites of Sciuridae (Ferris, 1919; Kim, 2006). These lice taxa were not previously recorded by Viljoen (1977c), but *W. paraxeri* was previously recorded from *P. cepapi* in Namibia, while *E. heliosciuri* was recorded from *P. cepapi* at an unknown locality (Johnson, 1960; Durden and Musser, 1994). Thus far, the distribution of *E. heliosciuri* includes Angola, Democratic Republic of Congo, Kenya, Liberia, Tanzania and Uganda (Durden and Musser, 1994). The present study is the first record of *W. paraxeri* and *E. heliosciuri* on *P. cepapi* in South Africa and thereby documents the most southern locality for the two louse species in Africa. It is interesting to note that the body size of *J. heliosciuri* is almost double the size (adult males measure 1·24 mm and adult females 1·74 mm) of *W. paraxeri* (0·55 and 0·68 mm, respectively) and *E. heliosciuri* (0·59 and 0·8 mm, respectively). Further, in the present study, *W. paraxeri* and *E. heliosciuri* were attached to the base of the hair shaft at the skin. The absence of the two louse species in the study by Viljoen (1977c) might be due to several reasons. In the present study, the squirrels were systematically examined under a stereomicroscope, while in the previous study, squirrels were most likely brushed (although no information regarding parasite removal is given). It is also possible that the lice were absent at the three sampling localities studied by Viljoen, (1977c).

The presence of two or three louse species is not uncommon for tree squirrels (Kim, 2006; Durden et al. 2024). For example, Pung et al. (2000) recorded three louse species on the southern flying tree squirrel (*Glaucomys volans*) in the USA, while Romeo et al. (2013) recorded two louse species on the Eurasian red tree squirrel (*Sciurus vulgaris*) in Italy and France. In both instances, the lice species belonged to different families. The three louse species in the present study were all relatively common (each occurred on more than 50% of the squirrels and co-occurred at least five localities). However, it is evident that two of the three louse species preferred distinct body regions. This, together with the fact that the two smaller species occur at the hair base where they grasp under the hairs (Durden et al. 2024), while *J. heliosciuri* was usually found further along the hair shaft away from the skin, which may facilitate the co-occurrence of multiple louse species on a single host. This pattern aligns with the concept of spatial niche partitioning (Chase and Leibold, 2012) and has been recorded in other parasite-host systems (Barnard et al. 2015; Fernández-González et al. 2015; Stefan et al. 2015).

*Rhipicephalus* cf. *zambeziensis* was the most common and widespread tick species on *P. cepapi. Rhipicephalus zambeziensis* was previously recorded on *P. cepapi* in South Africa (Horak et al. 2018). Similarly, the presence of *R. simus, R. theileri* and *H. zumpti* on *P. cepapi* is supported by Viljoen (1977c) and Horak et al. (2018). In the present study, only tick larvae and nymphs were recorded on *P. cepapi*. These life stages have fewer discerning morphological features and are thus more difficult to identify to species level (Walker et al. 2000). This is especially relevant for African species in the genus *Rhipicephalus*. To obtain conclusive evidence on the taxonomic status of the tick species, future studies should focus on a more comprehensive sampling of the ticks throughout their range in South Africa and Africa and should also include broad-scale molecular characterization of all the species known to be part of species complexes. Nonetheless, Hoogstraal and El Kammah (1974) noted that both adult and immature life stages of *H. zumpti* can occur on sciurid species and especially squirrels within the genus *Paraxerus*. In this study, however, adult *H. zumpti* were absent

Fleas and mites were less common on *P. cepapi*. The presence of *Xenopsylla* fleas on *P. cepapi* is in agreement with previous studies that recorded *X. brasiliensis* (Haeselbarth et al. 1966; Viljoen, 1977c) and *X.* z*umpti* (Haeselbarth et al. 1966) on *P. cepapi* in South Africa. The presence of *E. transvaalensis* (Hirstionyssinae) is in agreement with the study by Viljoen (1977c). At the time of the latter study, the particular mite was classified in the genus *Hirstionyssus* and referred to as *Hirstionyssus transvaalensis*. Fleas and mesostigmatic mites are both nest parasites with most life stages occurring in the host’s nest (Lehane, 2005; Dowling, 2006). As such, the diversity and abundance of fleas and mites are often related to the microclimatic conditions in the nest (Moreno et al. 2009). *P. cepapi* is known for frequently cleaning nest holes by removing and replacing the nesting material (Thorington et al. 2012), and this behaviour may create unfavourable conditions for fleas and mites (Bush and Clayton, 2018). Other contributing factors include auto- and allogrooming between conspecifics, a frequent practice for *P. cepapi* (Viljoen, 1977a; Hawlena et al. 2007; Moreno et al. 2013), and the incorporation of aromatic plants as nest material. Although there is no evidence yet as to the specific type of plant materials *P. cepapi* uses, besides leaves and grass, studies of other host-parasite systems (e.g. passerine birds such as the European Starling and its parasite load) have shown that the use of aromatic plants as part of the nesting material can act as a natural parasite repellent (Clark and Mason, 1985; Yang et al. 2020). Furthermore, the opportunistic sampling approach that was used in the study most probably biased the flea abundances. Fleas are known to abandon dead hosts as soon as the host’s body temperature begins to decrease (Russell, 1913; Westrom and Yescott, 1975). Any delay in placing a dead animal in a sample collection bag will allow fleas to leave the host and will bias the flea count. It is recommended that future studies adopt a standardized sampling approach that includes equal sample sizes per locality, a live-trapping approach and total ectoparasite counts.

Chiggers were the most speciose group of parasites on *P. cepapi*. Similar to previous rodent-chigger studies in South Africa (Fagir et al. 2014; Stevens et al. 2022; Smith et al. 2023), the ear was the preferred attachment site in the present study. Viljoen (1977c) previously identified chiggers on *P. cepapi* to genus level as a species similar to *Schoengastia.* Besides this record, there were no other chigger records associated with *P. cepapi* up to the present time, as well as very few species records associated with squirrels belonging to the genus *Paraxerus* in general. According to Vercammen-Grandjean (1958a, 1958b, 1965), four species (*Mictrotrombicula becquaerti, Mictrotrombicula paraxeri, Schoengastia katangae* and *Schoutedenichia paraxeri*) were previously recorded on the subspecies *P. c. quotus*, while *Schoutedenichia lumsdeni* was described from a host identified as a *Paraxerus* species from the Kruger National Park, South Africa. One new species, *Walchia africaeaustralis*, was recently described based on the material presented in Stekolnikov et al. (2025). As such, the present study provides new host records for eight of the nine recorded chigger species. Six of these species (*Ascoschoengastia ueckermanni, Herpetacarus decasetosus, Herpetacarus octosetosus, Microtrombicula graphiuri, Microtrombicula squirreli* and *Schoutedenichia morosi*) were previously recorded on rodents in South Africa (Stekolnikov and Matthee, 2019; Matthee et al. 2020; Stevens et al. 2022) and may therefore represent the chigger fauna characteristic of this region. *Microtrombicula polymorpha* is herein recorded in South Africa for the first time. This species was previously reported from two localities in DR Congo on three species of birds (Vercammen-Grandjean, 1965). Identification of *Hypotrombidium* sp. to the species level requires a revision of this genus in South Africa.

In the present study, *S. cepapi* was a very common and widespread nematode species of *P. cepapi*. This nematode was primarily recorded in the caecum, followed by the large intestine. Viljoen (1977b) recorded a different *Syphatineria* species, *Syphacia paraxeri* (at that stage still in the genus *Syphacia*), on *P. cepapi* at five localities in South Africa. However, it is possible that the identification of the *Syphatineria* specimens in Viljoen’s study was incorrect, given that, firstly, oxyurid pinworms are host specific, meaning they only occur in one (type) host (Sorci et al. 1997), and the ‘type host’ for *S. paraxeri* is *P. palliatus* (Sandground, 1933). Secondly, subsequent to Viljoen’s study (1977b), Hugot (1981) described *S. cepapi* from specimens obtained from one locality (Pretoria) in South Africa. The present study, therefore, provides new locality records for *S. cepapi* in South Africa. Transmission between hosts occurs directly through ingestion of eggs during grooming or coprophagy (Anderson, 1988, 2000). The relatively high prevalence of *S. cepapi* in *P. cepapi* is not unexpected given that the squirrel lives in small family groups that frequently partake in allogrooming (Viljoen 1977a).

The morphological characteristics of the *Strongyloides* species recorded in *P. cepapi* are not in agreement with any of the known *Strongyloides* species that have been documented for Africa (*S. fuelleborni* and *S. stercoralis*) and even South Africa (*S. ratti*) (Schär et al. 2013; Viney and Lok, 2015; Julius et al. 2017). Species–specific differences were observed in the length of several morphological features, such as the tail length and oesophagus length (Speare, 1986). Further, the twisting ovary and intestine that are present in *S*. cf *robustus* is absent in *S. stercoralis* and *S. ratti* (Little, 1966). Apart from the differences in measurements, the above-mentioned *Strongyloides* species exhibit different host preferences compared to *S*. cf *robustus*. For example, *S. fuelleborni* primarily occurs in African primates with the possibility of infecting humans (Linstow, 1905), while *S. stercoralis* occurs in humans (Genta, 1989) and *S. ratti* in rats (Sandground, 1925). It is interesting to note that most of the morphological characteristics of *S*. cf. *robustus* are, on average, shorter but closer to *S. robustus sensu stricto* from tree squirrels in the USA (Speare, 1986; Bartlett, 1995). However, the inclusion of molecular characterization, with more comprehensive taxonomic and geographic sampling of *Strongyloides,* is required to obtain a more robust species diagnosis.

The presence of only female *S*. cf. *robustus* nematodes is characteristic of the genus and supported by Bartlett (1995) and Viney and Lok (2015). The lower prevalence of *S*. cf. *robustus* compared to *S. cepapi* in *P. cepapi* is not uncommon, as a similar pattern was recorded in previous studies on rodents in South Africa (Julius et al. 2017) and elsewhere (Pung et al. 2000). The pattern might be due to life history differences, such as the transmission mode, between the two genera. In the case of *Strongyloides*, hosts become infected when the eggs found in the host faeces develop into infective L3 filariform stages via sexual and/or asexual development and penetrate the host skin when the host comes into contact with the faecal matter (Viney and Lok, 2015). This is in contrast to the life cycle of pinworms as mentioned above (Anderson, 2000).

Several of the identified parasites are of medical concern due to their zoonotic potential. For instance, *Xenopsylla* fleas are recognized vectors of *Yersinia pestis*, which is the causative agent of bubonic plague (Bitam et al. 2010). The immature stages of ticks belonging to the genera *Rhipicephalus* and *Amblyomma* have been implicated in the transmission of *Rickettsia africae*, the causative agent of African tick bite fever in humans (Kelly et al. 1996; Ledger et al. 2022). Additionally, several species in the genus *Strongyloides* have zoonotic potential, for example, *S. stercoralis* can infect humans and dogs and is the causative agent of strongyloidiasis (Jaleta et al. 2017), and *S. fuelleborni kellyi* is associated with swollen belly syndrome in humans (Bradbury, 2021).

The significant positive relationship between host body size and nematode infestation is consistent with the concepts that larger-bodied individuals are often older and, as such, have had more exposure time to parasites and provide more internal space for nematodes (Morand and Poulin, 2002; Kamiya et al. 2014; De Leo et al. 2016). This pattern has been observed in several rodent species, including the four-striped mouse (*Rhabdomys pumilio*) in the Western Cape Province, South Africa (Froeschke et al. 2013), the wood mouse (*Apodemus sylvaticus*) in southern England (Lewis et al. 2023), and in three rodent species (*A. sylvaticus, Apodemus flavicollis* and *Myodes glareolus*) in Serbia (Miljevic et al. 2022). Many monoxenous nematodes, and particularly pinworms (Oxyuridae) and threadworms (Strongylidae), have free-living life stages that infect hosts through ingestion during feeding and or allo- and autogrooming (Anderson, 1988, 2000; Morand et al. 2006). As such, larger individuals, who forage more and have lived longer, are more likely to encounter and ingest infective life stages (Morand and Poulin, 2002). Interestingly, nematode abundance was not significantly related to host sex, reproductive state, or their interaction. This is consistent with other studies that noted that sex-based and reproductive state differences in parasite load are not universally observed and can vary depending on host species, parasite taxon and ecological context (Zuk and Stoehr, 2009; Duneau and Ebert, 2012; Kiffner et al. 2013; Lo and Shaner, 2015; Lewis et al. 2023). The absence of a significant relationship between louse abundance and any of the host factors may be due to several reasons. Firstly, the methods used to kill the squirrels (culling and road kills) damaged portions of the host body, which may have influenced the louse counts, especially given the small size of two of the louse species. In addition, lice are transmitted through direct body contact, such as young suckling from their mothers and grooming between family members. This behaviour may influence significant relationships with host body size, sex and reproductive state. Similar to what was mentioned above, host body size, sex and reproductive state differences for lice infestations are not universal and depend, among others, on the host species, the age structure and season (Froeschke et al. 2013; Smith et al. 2023; Little et al. 2024)

The current study provides an update on the relatively rich ecto- and helminth parasite diversity associated with *P. cepapi* in South Africa. In addition, novel findings include new locality and/or country records for six of the recorded parasite species, and *P. cepapi* is a new host record for nine parasite species, and moreover, provides the first quantitative parasite data for *P. cepapi* in South Africa. Lastly, the study provides additional support for the role of host body size in shaping nematode infestations.

## Supporting information

Raubenheimer et al. supplementary material Raubenheimer et al. supplementary material

## Data Availability

All generated data for this study are included in this article. Additional data can be obtained from the corresponding author upon request.

## Supplementary material

The supplementary material for this article can be found at https://doi.org/10.1017/S0031182025101261.
