# Supplementary material for: The ectoparasites and gastrointestinal helminths associated with Smith’s bush squirrel (Paraxerus cepapi) in South Africa
Source: Parasitology. 2025 Nov 27;153(1):111–20. doi: 10.1017/S0031182025101261 (PMC13215735; doi:10.1017/S0031182025101261)
Supplement: Raubenheimer et al. supplementary material [file S0031182025101261sup001.docx]

**Supplementary Table 1.** Ectoparasite- and nematode species recorded on Paraxerus cepapi (n=94) in the Savanna biome, South Africa (2020-2024).

| **Taxon** | **Order** | **Suborder** | **Family/subfamily** | **Species** |
| --- | --- | --- | --- | --- |
| **Lice** | Phthiraptera | Anoplura | Polyplacidae | *Johnsonpthirus heliosciuri* Benoit, 1961 |
|  |  |  | Enderleinellidae | *Werneckia paraxeri* Werneck, 1947 |
|  |  |  |  | *Enderleinellus heliosciuri* Ferris, 1919 |
|  |  |  |  |  |
| **Ticks** | Ixodida |  | Ixodidae/Amblyomminae | *Amblyomma* sp. |
|  |  |  | Ixodidae/Haemaphysalinae | *Haemaphysalis elliptica/leachi* group |
|  |  |  |  | *Haemaphysalis zumpti* Hoogstraal & El Kammah 1974 |
|  |  |  | Ixodidae/Rhipicephalinae | *Rhipicephalus* cf. *simus* |
|  |  |  |  | *Rhipicephalus* cf. *theileri* |
|  |  |  |  | *Rhipicephalus* cf. *zambeziensis* |
|  |  |  |  |  |
| **Fleas** | Siphonaptera |  | Xenopsyllidae | *Xenopsylla* sp. |
|  |  |  |  |  |
| **Mites** | Mesostigmata |  | Laelapidae/Hirstionyssinae | *Echinonyssus* *transvaalensis* (Paolo, 1969) |
|  |  |  |  |  |
|  | Trombidiformes | Prostigmata | Trombiculidae/Trombiculinae | *Ascoschoengastia ueckermanni* Stekolnikov & Matthee, 2019 |
|  |  |  |  | *Microtrombicula* *polymorpha* (Vercammen-Grandjean, 1965) |
|  |  |  |  | *Herpetacarus decasetosus* Stekolnikov & Matthee, 2019 |
|  |  |  |  | *Herpetacarus octosetosus* Stekolnikov & Matthee, 2019 |
|  |  |  |  | *Hypotrombidium* sp. |
|  |  |  |  | *Microtrombicula* *graphiuri* Stekolnikov & Matthee, 2019 |
|  |  |  |  | *Microtrombicula* *squirreli* Stekolnikov, 2018 |
|  |  |  |  | *Schoutedenichia* *morosi* Vercammen-Grandjean, 1958 |
|  |  |  | Trombiculidae/Gahrliepiinae | *Walchia* *africaeaustralis* Stekolnikov, 2025 |
|  |  |  |  |  |
| **Nematodes** | Rhabditida |  | Strongylidae | *Strongyloides* cf. *robustus* |
|  | Oxyurida |  | Oxyuridae | *Syphatineria* *cepapi* Hugot, 1981 |

**Supplementary Table 2.** Prevalence (%) of ectoparasites and nematodes per sampling locality recorded on Paraxerus cepapi (n=94) in the Savanna biome, South Africa (2020-2024).

| **Taxon/Species** | **Groot Marico** | **Vaalwater** | **Marken** | **Alldays** | **Musina** | **Hoedspruit rural** | **Hoedspruit natural** | **Bushbuckridge** | **Total number of localities** |
| --- | --- | --- | --- | --- | --- | --- | --- | --- | --- |
| **Lice** |  |  |  |  |  |  |  |  |  |
| *Johnsonpthirus heliosciuri* | 66.67 | 100.00 | 30.77 | 71.43 | - | 95.00 | 73.91 | 50.00 | **7** |
| *Werneckia paraxeri* | - | - | 7.69 | 57.14 | - | 92.50 | 78.26 | 50.00 | **5** |
| *Enderleinellus heliosciuri* | - | - | 15.38 | 28.57 | - | 72.50 | 69.57 | 50.00 | **5** |
|  |  |  |  |  |  |  |  |  |  |
| **Ticks** |  |  |  |  |  |  |  |  |  |
| *Amblyomma* sp. | - | - | - | - | - | 2.50 | 4.35 | - | **2** |
| *Haemaphysalis elliptica/leachi* group | 33.33 | - | - | - | - | ­ | - | - | **1** |
| *Haemaphysalis zumpti* | 33.33 | - | - | 14.29 | - | 7.50 | 14.29 | - | **4** |
| *Rhipicephalus* cf. *simus* | ­ | - | - | - | - | 5.00 | - | - | **1** |
| *Rhipicephalus* cf. *theileri* | 33.33 | - | 15.38 | - | - | ­ | - | - | **2** |
| *Rhipicephalus* cf. *zambeziensis* | 66.67 | - | 46.15 | 100.00 | 100.00 | 15.00 | 47.83 | 100.00 | **7** |
| Unknown | - | - | - | - | 25.00 | - | - | - | **1** |
|  |  |  |  |  |  |  |  |  |  |
| **Fleas** |  |  |  |  |  |  |  |  |  |
| *Xenopsylla* sp. | - | - | - | - | - | 5.00 | - | - | **1** |
|  |  |  |  |  |  |  |  |  |  |
| **Mites** |  |  |  |  |  |  |  |  |  |
| *Echinonyssus* *transvaalensis* | - | - | 7.69 | - | - | - | - | - | **1** |
|  |  |  |  |  |  |  |  |  |  |
| **Chiggers** |  |  |  |  |  |  |  |  |  |
| *Ascoschoengastia ueckermanni* | - | - | - | - | - | 2.50 | 4.35 | - | **2** |
| *Microtrombicula polymorpha* | - | - | - | - | 25.00 | 2.50 | 4.35 | - | **3** |
| *Herpetacarus decasetosus* | - | - | - | - | - | - | 4.35 | - | **1** |
| *Herpetacarus octosetosus* | - | - | - | - | - | 7.50 | - | - | **1** |
| *Hypotrombidium* sp. | - | - | - | - | 50.00 | - | 8.69 | - | **2** |
| *Microtrombicula* *graphiuri* | 33.33 | - | - | - | - | 55.00 | 13.04 | - | **3** |
| *Microtrombicula* *squirreli* | - | - | - | - | 50.00 | 37.50 | 17.39 | 50.00 | **4** |
| *Schoutedenichia* *morosi* | - | - | - | - | - | 10.00 | - | - | **1** |
| *Walchia* *africaeaustralis* | - | - | - | - | - | 2.56 | - | - | **1** |
|  |  |  |  |  |  |  |  |  |  |
| **Nematodes** |  |  |  |  |  |  |  |  |  |
| *Strongyloides* cf. *robustus* | - | - | - | - | - | 38.46 | 18.18 | - | **2** |
| *Syphatineria* *cepapi* | 100.00 | - | 100.00 | 100.00 | 50.00 | 97.44 | 90.91 | 100.00 | **7** |
| **Cestodes** |  |  |  |  |  |  |  |  |  |
| Unknown | - | - | 7.69 | - | - | 2.56 | - | - | **2** |
| **Total number of species** | **7** | **1** | **8** | **6** | **6** | **18** | **14** | **6** |  |
